# Machine Learning and Imputation to Characterize Human Norovirus Genotype Susceptibility to Sodium Hypochlorite

**DOI:** 10.1007/s12560-024-09613-3

**Published:** 2024-09-11

**Authors:** Allyson N. Hamilton, Flor Maes, Génesis Yosbeth Chávez Reyes, Giselle Almeida, Dan Li, Mieke Uyttendaele, Kristen E. Gibson

**Affiliations:** 1 Department of Food Science, Center for Food Safety, University of Arkansas System Division of Agriculture, 1371 West Altheimer Dr, Fayetteville, AR 72704 USA; 2 Food Microbiology and Food Preservation Research Unit, Department of Food Technology, Safety and Health, Faculty of Bioscience Engineering, Ghent University, 9000 Ghent, Belgium; 3 Department of Food Science & Technology, Faculty of Science, National University of Singapore (NUS), Singapore, 117542 Singapore; 4 BESTMIX® Software, Vlaanderen, Maldegem, Belgium; 5 Steuben Foods Inc., Bozeman, Montana United States; 6 Arkansas Children’s Hospital, Little Rock, Arkansas United States

**Keywords:** Human norovirus, HuNoV, Bleach, Sodium hypochlorite, Multi-genotype, NaOCl

## Abstract

Human norovirus (HuNoV) is the leading cause of foodborne illness in the developed world and a major contributor to gastroenteritis globally. Its low infectious dose and environmental persistence necessitate effective disinfection protocols. Sodium hypochlorite (NaOCl) bleach is a widely used disinfectant for controlling HuNoV transmission via contaminated fomites. This study aimed to evaluate the susceptibility of HuNoV genotypes (*n* = 11) from genogroups I, II, and IV to NaOCl in suspension. HuNoV was incubated for 1 and 5 min in diethyl pyrocarbonate (DEPC) treated water containing 50 ppm, 100 ppm, or 150 ppm NaOCl, buffered to maintain a pH between 7.0 and 7.5. Neutralization was achieved by a tenfold dilution into 100% fetal bovine serum. RNase pre-treatment followed by RT-qPCR was used to distinguish between infectious and non-infectious HuNoV. Statistical methods, including imputation, machine learning, and generalized linear models, were applied to process and analyze the data. Results showed that NaOCl reduced viral loads across all genotypes, though efficacy varied. Genotypes GI.1, GII.4 New Orleans, and GII.4 Sydney were the least susceptible, while GII.6 and GII.13 were the most susceptible. All NaOCl concentrations above 0 ppm were statistically indistinguishable, and exposure duration did not significantly affect HuNoV reduction, suggesting rapid inactivation at effective concentrations. For instance, some genotypes were completely inactivated within 1 min, rendering extended exposure unnecessary, while other genotypes maintained the initial concentration at both 1 and 5 min, indicating a need for longer contact times. These findings underscore the critical role of HuNoV genotype selection in testing disinfection protocols and optimizing NaOCl concentrations. Understanding HuNoV susceptibility to NaOCl bleach informs better disinfection strategies, aiding public health and food safety authorities in reducing HuNoV transmission and outbreaks.

## Introduction

Human norovirus (HuNoV) is the leading cause of viral gastroenteritis globally, contributing significantly to morbidity and mortality. It was responsible for 242,996 (*95% UI*: 57,060–548,907) global deaths in 2019, is the primary cause of foodborne illnesses in the United States, and accounts for one-fifth of all diarrhea cases worldwide (Lopman et al., 2016; Zhang et al., 2022). The global age-standardized death rate (ASDR) is 1.86 (*95% UI*: 0.36–4.16), but for certain groups of people and within certain regions, it is much higher; for example, for individuals older than 70 years, the ASDR is 11.81 (*95% UI*: 1.28–29.54) (Zhang et al., 2022).

HuNoV infection typically manifests as acute gastroenteritis with symptoms including watery diarrhea, vomiting, nausea, and abdominal cramps. Severe dehydration can occur, especially in vulnerable populations such as the elderly, immunocompromised individuals, and young children. Despite the lack of specific antiviral treatments, supportive care focused on preventing dehydration is crucial (Hayashi et al., 2024; Zhang et al., 2022).

HuNoVs are non-enveloped, icosahedral viruses with a genome comprising single-stranded, positive-sense RNA organized into three open reading frames (ORFs). ORF1 encodes non-structural proteins, including a protease and RNA-dependent RNA polymerase, while ORF2 and ORF3 encode the major and minor capsid proteins, VP1 and VP2, respectively. The VP1 protein has two major domains: the shell (S) domain and the protruding (P) domain, responsible for receptor binding and immunogenicity (Hardy, 2005; Choi et al., 2008).

The resilience of HuNoVs complicates efforts to control their spread. Noroviruses are classified into 10 genogroups (GI–GX) and 49 genotypes based on the amino acid sequence of the capsid, with strains from genogroups GI, GII, and GIV historically affecting humans (Chhabra et al., 2019). To a lesser extent, GVIII (previously classified as GII.18) and GIX (formerly GII.15) have also been associated with human infections (Chhabra et al., 2019; Okada et al., 2005). As documented in outbreaks and analyses of surveillance data over the past 20 years, GII.4 is the predominant genotype causing infection in humans (Bull et al., 2006; Faarahmand et al., 2022; Siebenga et al., 2009; Zhang et al., 2024). Moreover, the GII.4 genotype has caused all major HuNoV pandemics due to its wide histo-blood group binding capabilities and high mutation rates (Buesa and Rodríguez-Díaz, 2016; Liang et al., 2021). New GII.4 strains emerge approximately every 2 to 3 years, increasing infections and posing significant public health challenges (Liang et al., 2021).

HuNoV spreads through direct interpersonal contact, contaminated food or contaminated water, and from surfaces in the environment that carry the virus, all of which involve transmission from fecal or vomit particles (Chandran and Gibson, 2024). Outbreaks often occur in settings involving food preparation, emphasizing the importance of food worker hygiene. HuNoV’s public health impact is driven by the sheer number of cases, leading to substantial healthcare costs and economic burdens (Scallan et al., 2011; Chandran and Gibson, 2024).

Due to their persistence in the environment and resistance to common disinfectants, effective inactivation protocols for HuNoVs are essential. Hypochlorite bleach is one of the most effective disinfectants and is widely used in the food industry (Cook et al., 2016). However, different HuNoV genotypes may exhibit varying susceptibilities to bleach, impacting the efficacy of disinfection strategies. Studies indicate variable resistance of HuNoV genotypes to alcohol-based disinfectants (Ettayebi et al., 2022; Park et al., 2016), suggesting potential differences in susceptibility to hypochlorite bleach as well. For instance, Tung et al. (2013) previously reported on the differences in log reduction of HuNoV GII.2 (Snow Mountain strain) and GII.4 when exposed to 1,000 ppm sodium hypochlorite (pH 7.3) for 30 s. Specifically, the study authors reported a ~ 4.5 log reduction for GII.4 while GII.2 was reduced by less than 0.5 log (Tung et al., 2013). In addition, Dunkin et al. (2017) observed significant differences in HuNoV GI.3 and GII.2 log reduction in chlorinated postharvest leafy green wash water suggesting HuNoV genogroup-dependent resistance to free chlorine (FC). Lastly, Costantini et al. (2018) utilized the human intestinal enteroid model (Ettayebi et al., 2016) to evaluate the inactivation of 3 GII.4 strains by chlorine bleach and observed complete log reduction at 50 ppm after 1 min. However, comparison of multiple HuNoV genotypes from within each genogroup for susceptibility to sodium hypochlorite in suspension has not been published, to the authors’ knowledge.

Therefore, this study aims to compare the susceptibility of a comprehensive set of HuNoV genotypes to sodium hypochlorite. By examining eleven HuNoV genotypes from genogroups I, II, and IV, the study assesses their inactivation levels when exposed to 50 ppm, 100 ppm, and 150 ppm bleach solutions for 1 and 5 min. Subsequent RNase pre-treatment, RNA extraction, and RT-qPCR analysis will determine the level of viral inactivation. Understanding the differential susceptibility of HuNoV genotypes to sodium hypochlorite will provide valuable insights for public health and food safety interventions.

## Materials and Methods

### Preparation of the Stool Samples

Stool samples containing HuNoV were preserved in PBS at a ratio of 1:10 and stored at − 80 °C. Upon thawing at ambient temperature, the samples underwent vortexing to dissociate virus particles from fecal matter. This was followed by centrifugation at 3000 × g (4000 rpm) for 10 min at 10 °C to clarify the samples. The resulting supernatant, containing the virus particles, was then transferred into 1.5 mL centrifuge tubes by aliquoting into 100 µL volumes per tube and stored at − 80 °C for future experiments. Details of HuNoV genotypes utilized in this study are presented in Table 1. All HuNoV samples were kindly provided by Dr. Jan Vinjé, Director of the Calicivirus Laboratory at the U.S. Centers for Disease Control and Prevention (Atlanta, GA, USA).

**Table 1:** Genotype-Specific evaluation of bleach concentrations (ppm). Genotype:bleach concentration pairs marked with ✓ indicate evaluation in the original dataset

Genotype	Concentrations of Bleach Evaluated (ppm)			
	0	50	100	150
GI.1	*✓*		*✓*	
GI.5	*✓*		*✓*	*✓*
GI.6	*✓*		*✓*	*✓*
GII.3	*✓*	*✓*	*✓*	*✓*
GII.4 New Orleans	*✓*		*✓*	*✓*
GII.4 Sydney	*✓*		*✓*	*✓*
GII.6	*✓*	*✓*	*✓*	*✓*
GII.7	*✓*		*✓*	*✓*
GII.13	*✓*	*✓*	*✓*	*✓*
GII.16	*✓*	*✓*		*✓*
GIV	*✓*		*✓*	

## RNA Extraction

RNA was extracted using the Qlamp DNA Blood Minikit and buffer AVL with carrier RNA (Qiagen, Germantown, MD) as described previously for viral nucleic acid extraction (Lambertini et al., 2008; Gibson et al., 2012; Borchardt et al., 2023). Prior to extraction, carrier RNA (Qiagen) was prepared according to the manufacturer’s instructions by mixing it with buffer AVL (220 µL of buffer AVL, 6.16 µL of carrier RNA). Briefly, virus particles were lysed by adding 25 µL of protease to a 1.5 mL centrifuge tube followed by the addition of 200 µL of the HuNoV sample and 200 µL of the carrier RNA mixture. The tube was then sealed and mixed thoroughly by pulse-vortexing for 15 s, then incubated for 15 min at 56 °C in a Dual Dry Bath Incubator Heat Block (Fisher Scientific, Hampton, NH). Following incubation, the tube was briefly centrifuged to eliminate condensation from the inside of the lid. Then, 250 µL of 95–100% ethanol was added, pulse-vortexed again for 15 s, and left to stand at room temperature for 5 min. Another brief centrifugation was performed to remove any residual drops from the lid.

The entire lysate was then transferred from the tube to a Qlamp MinElute column placed within a collection tube and manufacturer’s instructions were followed hereafter. For RNA elution, 40 µL of buffer AE was added directly onto the membrane in the column, incubated for 5 min at room temperature, and then centrifuged at 20,000 × g for 1 min. This elution step was repeated an additional time, ultimately yielding approximately 80 µL of extracted RNA in buffer AE.

## RT-qPCR Analysis

Following RNA extraction, RNA concentration was quantified via one-step RT-qPCR using a Mastercycler® ep *realplex* (Eppendorf, Hauppauge, NY). Primers and probes for different norovirus genogroups were sourced from Integrated DNA Technologies (Coralville, IA) and Biosearch Technologies (Petaluma, CA), detailed in Table 2. Specifically, primers and probes targeting a conserved region between the ORF-1 and ORF-2 junctions were utilized. For GI, the Norwalk/68 virus (GenBank accession no. M87661) was employed, whereas for GII, the Camberwell virus (GenBank accession no. AF145896) was used. The primers and probes for genotype IV (GIV), developed by the CDC, were based on the Saint Cloud virus (GenBank accession no. AF414427) and similarly targeted the junction between ORF-1 and ORF-2, as described by Kageyama et al. (2003) and Trujillo et al. (2006).

**Table 2:** Primers and probe oligonucleotides used for RT-qPCR (Kageyama et al., 2003; Trujillo et al., 2006)

Genotype	Primer/Probe	Sequence (5′—3′)^a^	Sense^b^	Location^c^
GI	Cog 1 F primer	CGY TGG ATG CGL TTY CAT GA	+	5291
GI	Cog 1 R primer	CTT AGA CGC CAT CAT CAT TYA C	−	5375
GI	Ring 1A probe	FAM-AGA TYG CGA TCY CCT GTC CA-BHQ	+	5340
GI	Ring 1B probe	FAM^**d**^-AGA TCG CGG TCT CCT GTC CA-BHQ^**e**^	+	5340
GII	Cog 2 F primer	CAR GAR BCN ATG TTY AGR TGG ATG AG	+	5003
GII	Cog 2 R primer	TCG ACG CCA TCT TCA TTC ACA	−	5100
GII	Ring 2 probe	FAM-TGG GAG GGC GAT CGC AAT CT-BHQ	+	5048
GIV	Mon 4 F primer	TTT GAG TCY ATG TAC AAG TGG ATG C	+	718
GIV	Mon 4 R primer	TCG ACG CCA TCT TCA TTC ACA	−	815
GIV	Ring 4 probe	FAM-TGG GAG GGG GAT CGC GAT CT-BHQ	+	763

^**a**^ R = A or G, Y = C or T, N = any

^**b**^ + , virus sense; − , antivirus sense

^**c**^ Nucleotide positions were taken from reference HuNoV strains in genogroups GI (Norwalk virus 68 [Genbank accession no. M87661]), GII (Hawaii virus [GenBank accession no. U07611]), and GIV (Saint Cloud virus [GenBank accession no. AF414426])

^**d**^ FAM, 6-carboxyfluorescein reporter dye

^**e**^ BHQ, black hole quencher dye

The preparation of the RT-qPCR master mix, requiring 20 µL per well, varied according to the target genogroup and consisted of both constant and genogroup-specific components. Universally, each well included 5.5 µL of DEPC-treated water (Qiagen), 12.5 µL of QuantiTect Probe RT-PCR Master Mix (Qiagen), 0.25 µL of RT Mix (Qiagen), and 0.25 µL of RNasin Plus RNase Inhibitor (Promega Corporation, Madison, WI). Specific additions varied by genogroup: for GI, the mix was supplemented with 0.5 µL each of Cog 1F (50 µM) and Cog 1R primers (50 µM), achieving a primer concentration of 1.25 µM each. Additionally, 0.25 µL each of Ring 1A (10 µM) and 1B probes (10 µM) were added, resulting in a probe concentration of 0.125 µM each. For GII, the additions were 0.5 µL each of Cog 2F (50 µM) and Cog 2R primers (50 µM) and 0.5 µL of Ring 2 probe (10 µM), establishing respective concentrations of 1.25 µM and 0.25 µM. GIV utilized 0.5 µL each of Mon 4F (15 µM) and Mon 4R primers (15 µM) and 0.5 µL of Ring 4 probe (20 µM), resulting in final concentrations of 0.375 µM for the primers and 0.5 µM for the probe (Gibson et al., 2011; Trujillo et al., 2006).

Following master mix preparation, 20 µL were combined with 5 µL of extracted RNA in a 96-well plate. Plates were sealed with plastic film, ensuring careful handling to avoid contamination and film staining. Negative controls, consisting solely of master mix and bleach solution, were included to assess potential contamination. The thermal profile was uniform across genogroups, initiating with a 30-min segment at 50 °C for reverse transcription, followed by a rise to 90 °C to deactivate the reverse transcriptase and denature the DNA. The amplification process consisted of 45 cycles, each comprising a 15-s denaturation at 95 °C and a 1-min annealing/extension at 60 °C (Gibson et al., 2011).

## RT-qPCR Tests

Preliminary RT-qPCR testing was completed for all genotypes (Table 1) and used for constructing standard curves. Initially, 100 µL of each HuNoV sample was thawed, and RNA extraction was performed using the Qlamp DNA Blood Mini Kit and AVL buffer with carrier RNA (Qiagen) following previously described protocols. Subsequently, a tenfold serial dilution from − 1 to − 5 was prepared using DEPC-treated water. This dilution series was then subjected to RT-qPCR analysis, with each dilution run in triplicate on a microtiter plate.

## HuNoV Bleach Treatment

All 11 genotypes (Table 1) initially underwent treatment with 100 ppm bleach. Based on these initial outcomes, to efficiently utilize the limited samples available, subsequent tests employed either higher or lower bleach concentrations. Notably, when GII.16 exhibited no amplification at 100 ppm, additional evaluations at 50 ppm and 150 ppm were conducted.

Six vials were prepared at the start of each experiment to assess viral inactivation by bleach. The composition and treatment of these vials were as follows: a positive control containing 20 µL of the virus sample and 180 µL of DEPC-treated water, with no bleach added. A negative control containing 20 µL of bleach (150 ppm, 100 ppm, or 50 ppm) and 180 µL of DEPC-treated water, with no virus added. Four bleach treatment vials for each genotype, each containing 20 µL of the virus sample, 20 µL of bleach (150 ppm, 100 ppm, or 50 ppm), and 160 µL of DEPC-treated water.

All vials were brought to a final volume of 200 µL and incubated at room temperature. The incubation times were set at 1 min and 5 min for the bleach treatment vials, resulting in four treated vials: two for the 1-min incubation and two for the 5-min incubation. Following the incubation period, 20 µL from each bleach-treated vial was immediately neutralized by dilution into 180 µL of 100% FBS. This process yielded a total of six vials for further analysis: one positive control, one negative control, two bleach-treated vials with a 1-min incubation, and two bleach-treated vials with a 5-min incubation.

After the incubation and neutralization steps, all samples underwent RNase treatment (described below) followed by RNA extraction using the previously described method. The extracted RNA was analyzed using RT-qPCR as per the established protocol. This step confirmed the presence or absence of viral RNA, thereby allowing assessment of the efficacy of the bleach treatment in inactivating the virus.

## RNase Pre-Treatment

Following bleach treatment, RNA from compromised viruses may remain in the solution. To eliminate RNA not shielded by an intact viral capsid, an RNase treatment was employed. The treatment solution was prepared by combining 33 µL of DEPC-treated water, 15 µL of RNase ONE 10 × Reaction Buffer (Promega Corporation), and 1 µL of RNase ONE ribonuclease (Promega Corporation). This mixture was added to the sample vial, which was subsequently vortexed to ensure thorough mixing. The vial was then incubated for one hour at 37 °C to facilitate the RNase activity. Following incubation, 2 µL of RNasin® Plus RNase Inhibitor (Promega Corporation) was introduced to the vial to deactivate the RNase, ensuring preservation of intact viral RNA.

## Imputation of Ct Values

Two experimental trials, each with three replicates, were conducted for each combination of genotype, time, and bleach concentration. However, 2% of the cycle threshold (Ct) readings were missing, representing 0.3% of the total dataset. Due to the unavailability of additional virus samples, further experiments to obtain the missing data were not feasible. Consequently, imputation methods were employed to create a more complete dataset for subsequent analysis. The imputation was carried out using R (R Core Team, 2021) and incorporated several packages: *base*, *ggplot2* (Wickham, 2016), *tidyverse* (Wickham et al., 2019), *naniar* (Tierney and Cook, 2023), *simputation* (van der Loo, 2022), and *mice* (Buuren and Groothuis-Oudshoorn, 2011).

The nonparametric missing value imputation using a random forest was employed, which yielded a promising normalized root mean squared error (NRMSE) of 0.07. Despite this, manual inspection revealed that the random forest method produced unreasonable predictions, specifically for the GI.5 genotype. To address this issue, four additional imputation methods available within the *mice* package were compared: predictive mean matching (pmm), Bayesian linear regression (norm), random forest (rf), and classification and regression trees (cart). After thorough manual inspection, the cart method was determined to be the most effective. This method was implemented for the imputation of missing values. The fit and distribution of the imputed values demonstrate the efficacy of the cart method in maintaining the integrity of the dataset. This thorough approach ensures a robust and reliable dataset, facilitating accurate and meaningful downstream analysis (Chhabra et al., 2017).

## Generation of Standard Curves with Unknown Initial Viral Concentrations

Two experimental trials, each with three replicates, were conducted for each genotype. However, 18% of the cycle threshold (Ct) readings were missing, representing 4.5% of the total dataset. Due to the unavailability of additional virus samples, it was not feasible to perform further experiments to obtain the missing data. Consequently, imputation methods were employed to create a more complete dataset for generating standard curves. The imputation was carried out using R (R Core Team, 2021) and incorporated the same packages used in “Imputation of Ct values”.

Linear imputation resulted in extreme values rendering it incompatible for our dataset. Thus, nonparametric missing value imputation using a random forest (*with ntree* = 10000) was implemented, which yielded a low normalized root mean squared error (NRMSE) of 0.0002. Manual inspection confirmed that this method produced reasonable predictions for all genotypes. Therefore, the random forest method was adopted for the imputation of missing values.

With a complete dataset, the range of Ct values was used to create a key to assign values of initial concentration to the most concentrated sample, rounded to the nearest log genomic copies (gc) per reaction (Table 3). Subsequent dilutions of each genotype were assigned concentrations by reducing the concentration in a tenfold manner per tenfold dilution. Following these assignments, standard curves were generated. The slopes, intercepts, and *R*^2^ values of these standard curves are presented in Table 4.

**Table 3:** Initial concentration assignment key for all genotypes

Ct Range	Log(Genomic Copies/Reaction)
20.61–22.90	8
22.91–25.19	7
25.20–27.48	6
27.49–29.77	5
29.78–32.05	4
32.06–34.34	3
34.35–36.62	2
36.63–38.91	1

**Table 4:** Log10 dilution series of HuNoV stool samples and corresponding slopes

Genotype	Slope	Intercept	R^2^
GI.1	− 2.48	39.67	0.98
GI.5	− 2.91	39.95	0.97
GI.6	− 2.91	40.99	0.95
GII.3	− 1.51	38.36	0.92
GII.4 New Orleans	− 3.07	39.06	0.99
GII.4 Sydney	− 2.42	40.86	0.97
GII.6	− 3.60	40.75	0.99
GII.7	− 2.40	39.18	0.88
GII.13	− 3.18	39.76	1.00
GII.16	− 3.31	41.75	0.98
GIV	− 2.48	39.69	0.87

## Machine Learning-Based Prediction of Ratio Values

To predict the ratio values for each genotype, bleach concentration, and time point, including confidence intervals, machine learning techniques were utilized. The machine learning was carried out using R (R Core Team, 2021) and incorporated several packages: *base*, *ggplot2* (Wickham, 2016), *tidyverse* (Wickham et al., 2019), *caret* (Kuhn, 2008), *randomForest* (Breiman, 2001)*, gbm* (Ridgeway, 2019)*,* and *boot* (Canty and Ripley, 2024; Davison and Hinkley, 1997). The dataset was pre-processed to assure factors were converted, structured, and split into training and test sets. The training segment held 80% of the data points, and 20% were allocated to test, which delivered a representative sample for model validation.

Two machine learning models were trained: Random Forest (RF) and Gradient Boosting Machine (GBM). The RF model was configured with 500 trees to optimize performance, while the GBM model was trained with 500 trees, an interaction depth of 3, and a shrinkage rate of 0.1, incorporating fivefold cross-validation to enhance its robustness. Both models were evaluated using the root mean squared error (RMSE) metric, which was calculated by comparing the model predictions against the actual values in the test dataset.

Upon evaluation, the GBM model exhibited a lower RMSE (0.09) compared to the RF model (0.18), indicating superior predictive performance. The GBM model was selected as the best-performing model for subsequent predictions. Using the selected GBM model, predictions were generated for all unique combinations of genotype, time, and bleach concentration, whether present in the initial dataset or not. To provide a measure of uncertainty around these predictions, pseudo-confidence intervals were calculated. These intervals were derived by estimating the prediction error from the training data and applying a 1.96 multiplier to approximate the 95% confidence range. This approach ensured that the lower bounds of the confidence intervals were constrained to a minimum of 0 and the upper bounds to a maximum of 1, reflecting the possible range of the ratio values.

## Statistical Analysis

Statistical analyses were conducted to evaluate whether genotype, bleach concentration, or time significantly predicted the reduction in the HuNoV recovery ratio. The proportion recovered was calculated by dividing the genomic copies per reaction recovered post-treatment by the gc in the time-zero positive control. Initially, the data were analyzed using a linear model in R Studio (R Core Team, 2021). However, residual analysis indicated violations of the assumptions of normality and homoscedasticity. Furthermore, the sample size was insufficient to rely on the central limit theorem for approximating normality of sample means.

Given the non-normality, the data were re-analyzed using a generalized linear model (GLM) with quasibinomial errors to account for overdispersion, as indicated by a residual deviance greater than the residual degrees of freedom. Estimated marginal means were used to calculate the ratio means and their associated standard errors. Interaction effects were tested, but none were found to be significant. Statistical differences between treatments were assessed through multiple comparisons, and the results were visualized using a compact letter display.

The analysis was performed using R Studio (R Core Team, 2021) with several packages, including *base*, *ggplot2* (Wickham, 2016), *emmeans* (Length et al., 2021), *tidyverse* (Wickham et al., 2019), *ggpubr* (Kassambara, 2020), *gdata* (Warnes et al., 2022), *rstatix* (Kassambara, 2021), *lme4* (Bates et al., 2015), *lmertest* (Kuznetsova et al., 2017), *multcomp* (Hothorn et al., 2008), and *multcompView* (Graves et al., 2019).

## Results & Discussion

The primary objective of this study was to evaluate the susceptibility of various HuNoV genotypes to sodium hypochlorite in suspension, thereby contributing to the understanding of the efficacy of disinfection strategies. The results indicate bleach effectiveness as a disinfectant and highlight the differential susceptibility of HuNoV genotypes. These findings have implications for public health and food safety, particularly in settings where HuNoV outbreaks are common, such as food service environments and healthcare facilities.

**Fig. 1:**
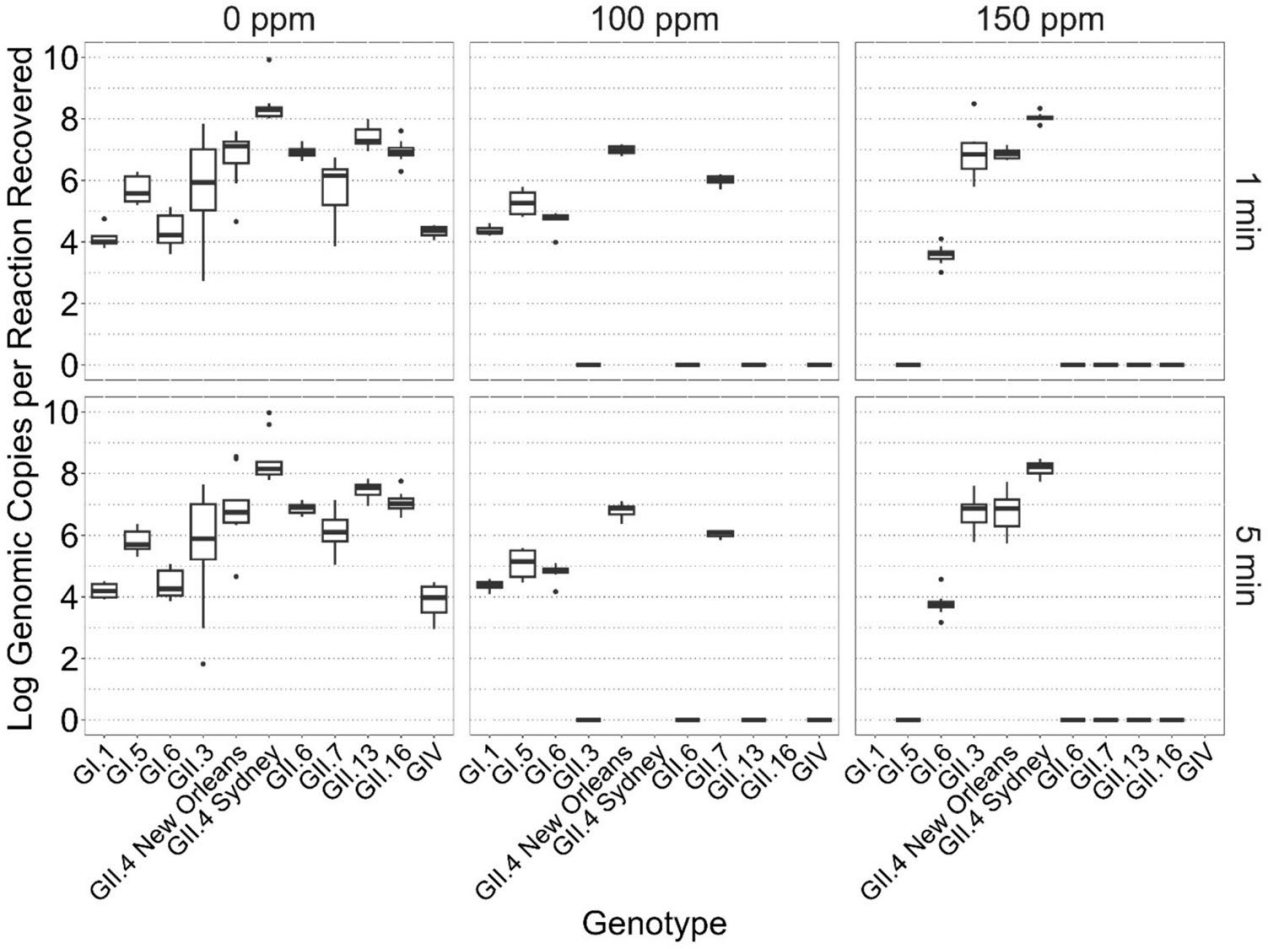
Boxplot of raw log genomic copies per reaction recovered, stratified by genotype, time, and concentration of bleach. Data for 50 ppm (GII.3, GII.6, GII.13, GII.16) not shown as all values were 0

**Fig. 2:**
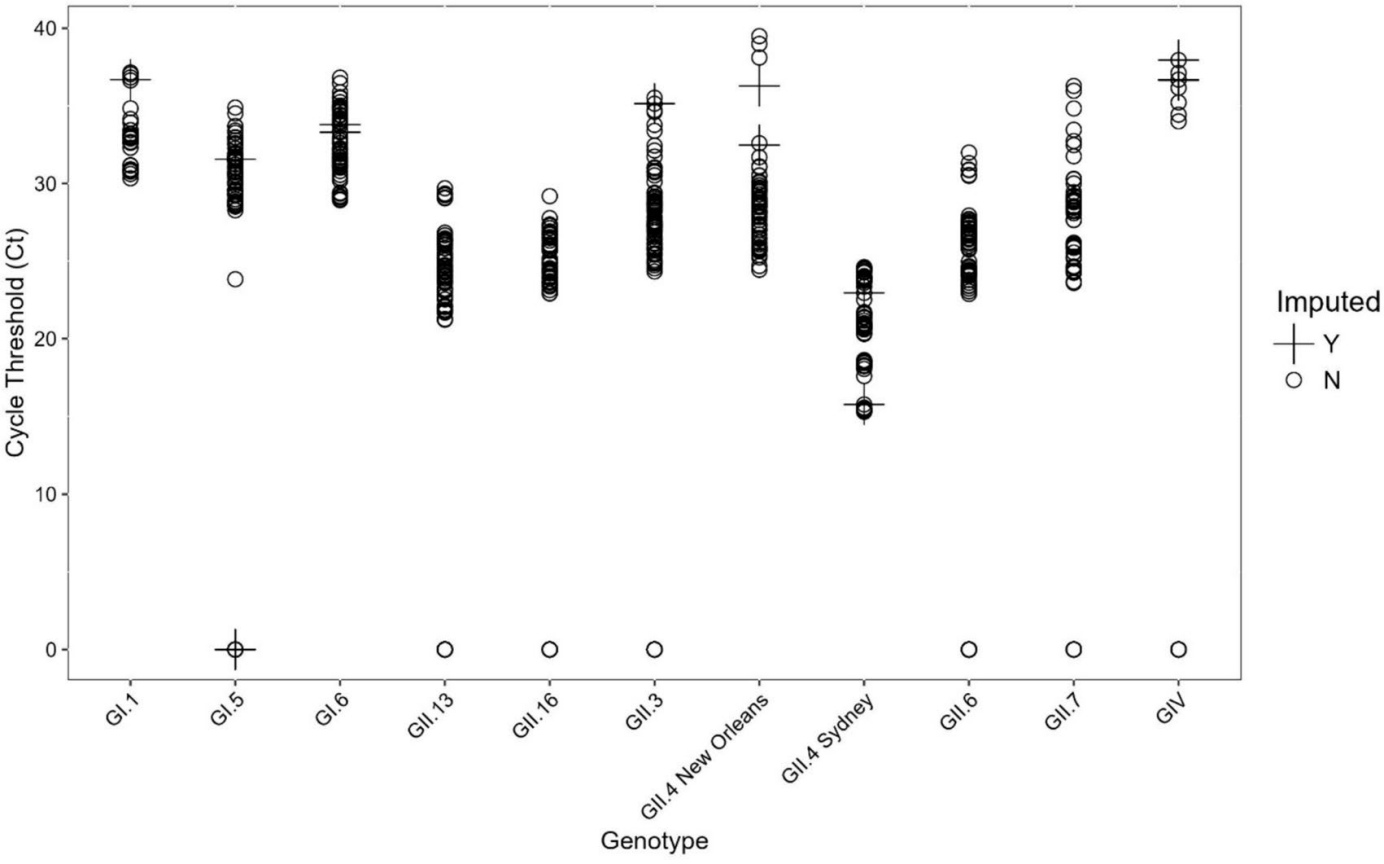
Results of classification and regression trees (cart) imputation of cycle threshold (Ct) data. Imputed data are represented by a “ + ” and original data are represented by a “o”

**Fig. 3:**
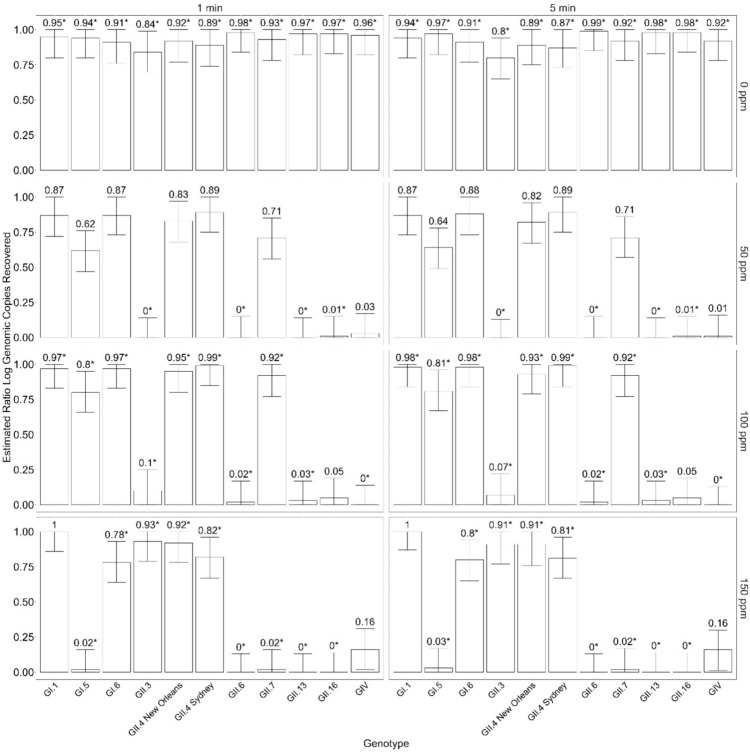
Machine learning-based predictions of the ratio of log genomic copies recovered to positive control, stratified by time and bleach concentration. Error bars represent pseudo-confidence intervals at the 95% level. Ratios indicated with an asterisk (*) next to the column label denote that the genotype:time:concentration_bleach combination was present in the original dataset and was not generated de novo by the machine learning model

All raw data are presented in Fig. 1, which illustrates the estimated log gc per reaction recovered, categorized by genotype, bleach concentration, and time. Data for 50 ppm (GII.3, GII.6, GII.13, GII.16) are not shown as all values were 0. This dataset was subjected to a process of imputation to address missing values (Fig. 2) and was further refined using techniques derived from GBM learning (Fig. 3). These steps were essential to ensure a complete and balanced dataset, suitable for subsequent statistical analysis using a GLM. The presence of zero values for all ratios at 50 ppm bleach concentration in the initial dataset represents a significant challenge. This absence of data complicates the estimation processes typically employed by machine learning models and explains some of the observed variability between the data for 50 ppm bleach and other concentrations, as detailed by Fávero et al. (2024).

The application of a GLM revealed no significant interaction effects between genotype, bleach concentration, or time. The time factor was not significant (*p* = 0.96), indicating no substantial influence of exposure duration on the recovery ratio. However, both genotype (*p* = 8.9E-5) and bleach concentration (*p* = 2.4E-7) were highly significant predictors of the recovery ratio, highlighting the pronounced impact of these variables on the estimated log gc per reaction recovered.

## Impact of Genotype on Virus Reduction

**Fig. 4:**
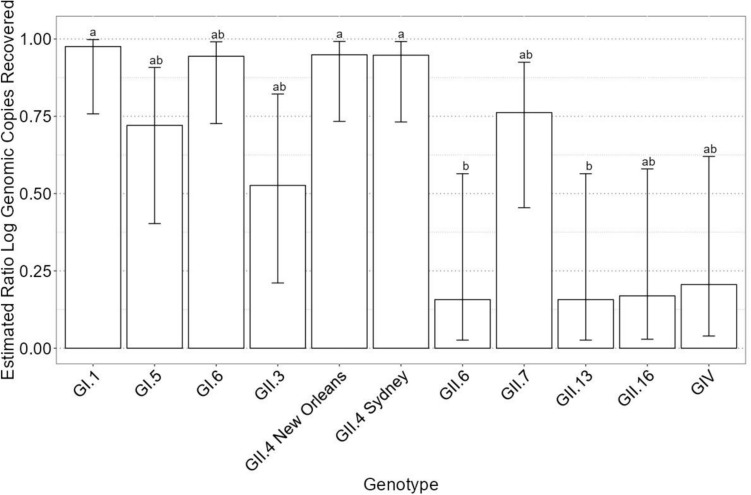
Generalized linear model with quasibinomial errors for the estimated ratio recovery of human norovirus per genotype. Compact letter format is used to designate statistical differences between treatments at *P* = 0.05. Error bars represent 95% confidence intervals

Figure 4 delineates the genotype-based differences in virus recovery after NaOCl treatment, categorizing the genotypes into three distinct statistical groups based on log gc remaining after treatment. Genotypes GI.1, GII.4 New Orleans, and GII.4 Sydney exhibited the highest recovery rates, showing minimal reductions in log gc, indicating a lower level of inactivation by bleach. In contrast, genotypes GI.5, GI.6, GII.3, GII.7, GII.16, and GIV showed intermediate levels of inactivation, reflecting moderate recovery rates. The lowest recovery rates were observed in GII.6 and GII.13, marking substantial reductions in log gc.

This stratification of virus inactivation highlights the inherent variability among HuNoV genotypes and suggests a varying resilience to bleach treatment. These results emphasize the need to consider genotype variations when devising and testing disinfection strategies. This research incorporated a broad representation of HuNoV strains from genogroups I, II, and IV, encapsulating a significant portion of the diversity present in circulating strains (Chandran and Gibson, 2024). Comparative studies, such as the investigation by Park et al. (2016), have also examined the efficacy of other disinfectants like ethanol. The study authors observed a reduction of only about 0.5-log in GII.4 New Orleans after a 1-min treatment with 70% ethanol, whereas GII.4 Sydney experienced a 2-log reduction under identical conditions. Additionally, genotypes GI.1, GI.6, GII.7, and GII.13 exhibited negligible inactivation from the same ethanol treatment (Park et al., 2016).

Relevant to the present study, Kadoya et al. (2022) observed that enhancing intrapopulation genetic diversity within two ancestral populations of an enteric RNA virus— rhesus rotavirus (RRV)—significantly impacted resistance to disinfection methods. Utilizing a series of experimental and control groups, Kadoya et al. (2022) conducted serial passaging of RRV strains and applied various concentrations of chlorine-based disinfectants. Through qPCR and next-generation sequencing techniques, it was observed that virus populations with elevated genetic diversity, particularly in non-synonymous mutations of the outer capsid protein gene, exhibited a markedly higher incidence of chlorine resistance. These insights affirm the complex interaction between virus genetic diversity and disinfection efficacy.

## Effect of Bleach Concentration on Virus Reduction

**Fig. 5:**
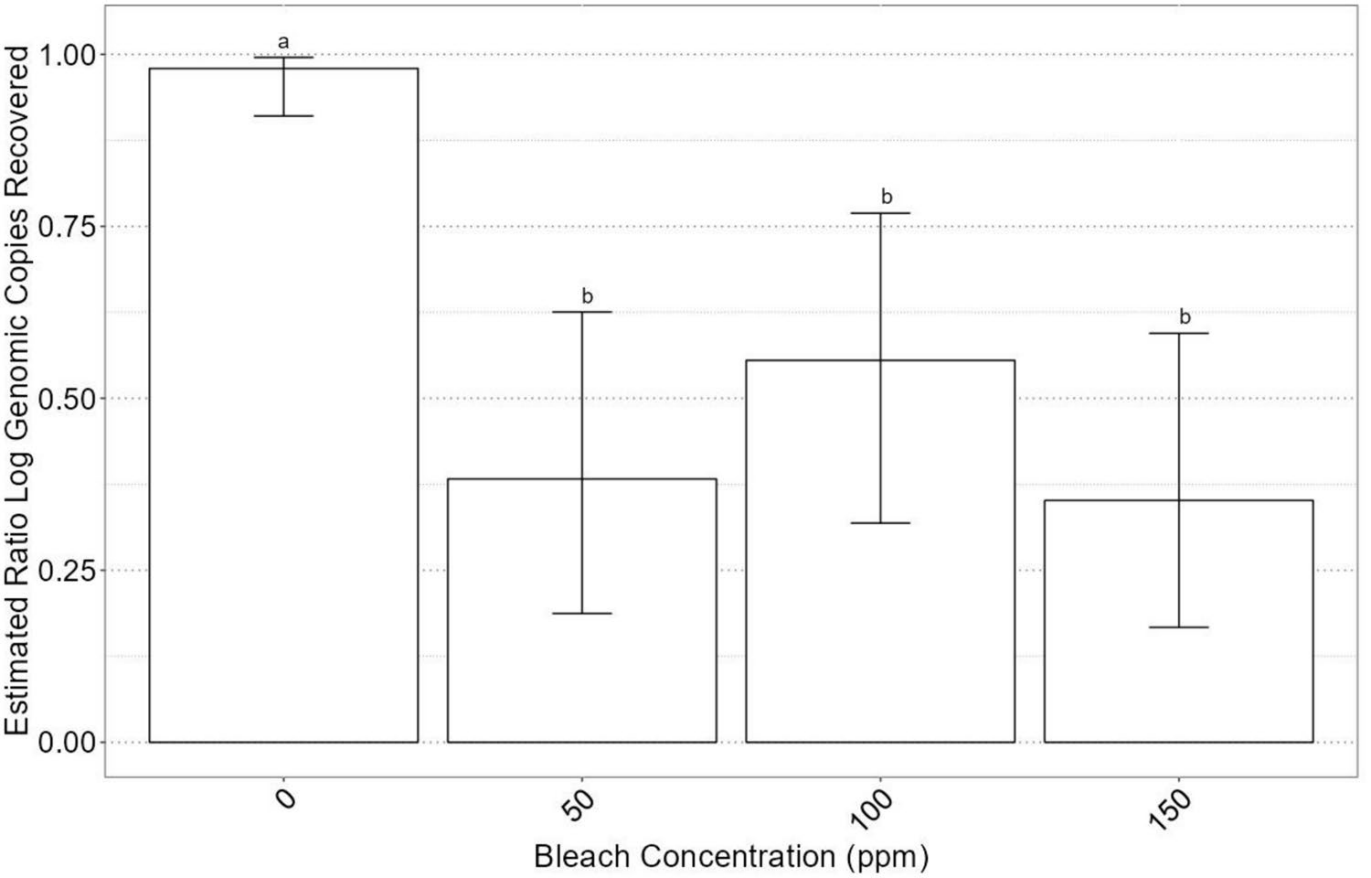
Generalized linear model with quasibinomial errors for the estimated ratio recovery of human norovirus per bleach concentration. Compact letter format is used to designate statistical differences between treatments at *P* = 0.05. Error bars represent 95% confidence intervals

Figure 5 shows the influence of NaOCl concentration on the recovery ratio of log gc of HuNoV. The results revealed two statistically significant tiers based on the presence or absence of bleach, and it was observed that all bleach concentrations above 0 ppm resulted in similar virus recovery (i.e., minimal change in log gc). The absence of significant differences among higher concentrations points to a threshold effect, wherein the incremental increase in bleach concentration beyond a certain point does not proportionally increase virus inactivation. This finding is crucial for the optimization of disinfection protocols, suggesting that lower concentrations of bleach may be adequately effective depending on genotype, thus potentially reducing both costs and environmental impact. However, this would heavily rely on the development of rapid genotyping techniques at the onset of an outbreak.

Escudero-Abarca et al. (2022) corroborated similar findings in their study, noting no significant differences in GII.4 Sydney reduction across bleach concentrations ranging from 100 to 5000 ppm in solution when exposed for 60 s with a “native soil load” (i.e. clarified stool sample soil load, ~ 2.5%). With the addition of more soil (5%), the efficacy of 100 ppm bleach against HuNoV was significantly reduced while exposure to 500 to 5000 ppm achieved the same level of reduction (~ 4.5 log gc). In additional experiments on surfaces with native soil loads (~ 2.5%), Escudero-Abarca et al. (2022) highlighted that while 100 ppm was less effective (~ 1 log gc reduction), no further reduction benefit was seen when comparing 500 ppm to 5000 ppm concentrations since ~ 4.5 log gc reduction was observed at all of these concentrations. These findings indicate that bleach concentration needs to be optimized between 100 and 500 ppm when native soil loads are present. Moreover, it would be prudent to optimize contact time, as all experiments were completed at 60 s (Escudero-Abarca et al., 2022).

In their review of chlorine-based surface disinfection efficacy, Gallandat et al. (2021) highlight the inconsistencies, vagueness, and lack of evidence-based protocols for pathogens associated with large outbreaks, such as HuNoV. For instance, the CDC (2011) recommends using a chlorine bleach solution with a concentration of 1,000 to 5,000 ppm or an EPA-registered disinfectant effective against norovirus, with a contact time of at least 5 min on surfaces. The lack of specificity regarding concentration and contact time, likely leads to significant bleach overuse and much practitioner confusion regarding contact time.

Additionally, virus aggregation has been proposed as a potential explanation for the complex behavior of viruses in the environment and their resistance to disinfectants (Clark, 1968; Gerba and Betancourt, 2017; Hoff and Akin, 1986). For instance, Mattle et al. (2011) highlighted that viruses in natural settings often exist as aggregates, yet recommended disinfectant use is typically determined based on more dispersed virus forms. Notably, the study revealed that MS2 aggregation led to a 2 to sixfold reduction in apparent inactivation rate constants, with larger aggregates and higher peracetic acid concentrations intensifying the inhibitory effect of aggregation on disinfection (Mattle et al., 2011).

Research into the inactivation mechanisms of HuNoV by FC, conducted by Nowak et al. (2011), concluded that the primary mode of inactivation is via virus capsid degradation, leading to the exposure of RNase-susceptible viral RNA and a consequent loss of infectivity. Notably, the loss of infectivity due to capsid degradation is not unique to HuNoV. Inactivation of enteroviruses by FC has also identified capsid damage as the predominant factor influencing virus inactivation (Alvarez and O’Brien, 1982; Nuanualsuwan and Cliver, 2003; Torrey et al., 2019; Wigginton et al., 2012).

Additionally, Nowak et al. (2011) demonstrated that surrogates might not be appropriate for disinfection efficacy studies; three non-epidemiologically linked human GII.4 noroviruses in dilute clinical samples showed similar reductions to each other but were ten times more resistant to virolysis than cultured Feline Calicivirus F-9 (FCV F-9). When FCV F-9 was present in dilute human GII.4 samples, it acquired increased resistance to virolysis approaching that of HuNoVs, highlighting the significant impact of matrix effects on viral persistence (Nowak et al., 2011).

The variability in susceptibility among different virus variants to FC treatment can be largely attributed to differences in the integrity of essential viral functions, specifically receptor binding and uncoating (Torii et al., 2022; Torrey et al., 2019). These findings suggest that the structural and functional robustness of viral proteins play a significant role in determining their vulnerability to disinfection processes (Torii et al., 2022; Torrey et al., 2019; Wigginton et al., 2012). Consequently, a nuanced understanding of these mechanisms is crucial for the development of effective virucidal agents and protocols.

Although RT-qPCR is a powerful technique for detecting HuNoV, it cannot distinguish between RNA from infectious and non-infectious viruses. However, preceding molecular amplification with an RNase pre-treatment has been employed to better differentiate infectious from non-infectious RNA virus particles post-treatment (Manuel et al., 2016; Montazeri et al., 2017). In this method, RNA from compromised viral capsids is degraded by RNase, allowing for the quantification of only intact (and presumably infectious) virus particles (Knight et al., 2012). This method, among many others, has been discussed at length by Monteiro and Santos (2017).

## Effect of Time on Virus Reduction

Despite the clear impact of bleach presence and the specific genotype on HuNoV inactivation, the duration of exposure (1 min vs. 5 min) did not significantly affect the reduction in viral load in this study. This may be because some genotypes were completely inactivated within 1 min, rendering extended exposure unnecessary, while others maintained their viral load at both 1 and 5 min, indicating a need for longer contact times. Nevertheless, these results suggest that the inactivation process may occur rapidly once an effective bleach concentration is reached, and that extending the exposure time does not confer additional benefits. This insight simplifies disinfection protocols by showing that short contact times are sufficient, provided the bleach concentration is adequately maintained.

In a detailed investigation of disinfection across different viruses, Shin and Sobsey (2008) used FC to assess the inactivation response of Norwalk virus (HuNoV GI.1), comparing it to coliphage MS2 and poliovirus 1 (PV1) under similar conditions in wastewater. The study authors demonstrated variances in virus inactivation rates that are dependent on exposure time to the disinfectant. Specifically, GI.1 and MS2 showed a rapid inactivation at lower chlorine concentrations, with MS2 achieving a 5 log reduction in infectivity within a mere 20 s and NV reaching a 2 log reduction within 3 min (Shin and Sobsey, 2008). In contrast, PV1 showed a slower response, requiring up to 10 min to achieve a 4 log reduction (Shin and Sobsey, 2008). These results suggest that HuNoV GI.1, despite previous studies indicating its high resilience to chlorine (Keswick et al., 1985), can be effectively inactivated within short time frames provided that the appropriate concentration and environmental conditions (such as pH and temperature) are maintained. This contrasts with the more extended exposure times required for PV1, reflecting intrinsic differences in virus structure and possibly outer protein compositions that influence their susceptibility to chlorine-based disinfection.

Regarding other HuNoV genotypes, Goda et al. (2022) found that HuNoV GII.4 and GII.2 exhibited a remarkably similar reduction (~ 3 log) from initial concentrations of 7 log when exposed to 200 ppm NaOCl for durations of 1, 5, 10, and 30 min in the absence of bovine serum albumin (BSA). In the presence of BSA, the reductions were also similar between all genotypes and times, but significantly lower, achieving less than a 0.5 log reduction in all instances (Goda et al., 2022). This indicates that the presence of BSA considerably hampers the effectiveness of NaOCl in reducing viral load, emphasizing the importance of environmental factors in the disinfection process.

## Challenges and Limitations

One of the major challenges encountered in this study was the variability in the detection of viral RNA across different genotypes and bleach concentrations, and the lack of non-zero data for 50 ppm bleach. This variability necessitated the use of sophisticated statistical and machine learning techniques to handle missing data and ensure robust analysis. The application of the GBM model proved effective in predicting missing values and providing confidence intervals for the data, thereby enhancing the study’s analytical rigor. However, a limitation of the study is that GBM-generated data was used for statistical analysis via GLM.

It is also important to acknowledge that the inactivation data pertain solely to HuNoV in suspension with native soil loads, rather than on surfaces. This distinction is important as the dynamics of virus inactivation can differ significantly between these two contexts, potentially affecting the generalizability of the results to real-world settings where surface contamination is common (Tung-Thompson et al., 2014). Furthermore, the impact of organic matter on the efficacy of sodium hypochlorite was not explicitly studied within this framework. While the experiments utilized clarified stool samples, which still contain residual particulates at approximately at 2.5% soil load (Escudero-Abarca et al., 2022), these do not fully replicate the complexity of environmental organic matter typically found in outbreak scenarios (Djebbi-Simmons et al., 2020). This factor could influence the effectiveness of disinfection practices, as organic matter can protect viruses from the action of disinfectants (Djebbi-Simmons et al., 2020). Moreover, subsequent to the introduction of samples into the chlorine solutions, the quantification of free chlorine levels was not conducted. It is conceivable that the chlorine demand exhibited variation across preparations derived from distinct stool samples. Further studies are required to quantify these effects more precisely, potentially drawing on existing research that attempts to characterize the remaining particulate matter in such biological samples.

Another limitation of the study is related to the standard curves used to estimate log gc for each HuNoV genotype (Table 4). Ideally, the change in cycle threshold during a tenfold dilution series should be approximately 3.32 cycles per dilution (Svec et al., 2015); however, the RNA extracted from the samples used in the present study resulted in 1.5 to 3.6 cycle difference per dilution. The efficiency of the RT-qPCR assay likely varied by stool sample even within genogroup for multiple reasons. First, standard curves were not generated based on a linear dsDNA standard but rather RNA extracted from stool samples which was subsequently subjected to reverse transcription followed by qPCR. The suboptimal change in Ct values is possibly due to the inefficiency of the reverse transcription step (Kelly et al., 2022), presence of PCR inhibitors (Gibson et al., 2012), and inefficiency of the selected primers and probes for the detection of certain genotypes (Bustin et al., 2009).

## Conclusions

This study provides a comprehensive analysis of the susceptibility of various HuNoV genotypes to sodium hypochlorite, revealing significant variability in bleach effectiveness as a disinfectant. The findings highlight the importance of considering genotype-specific responses in disinfection protocols and underscore the need for ongoing research to understand the genetic factors underlying these differences. By optimizing bleach concentrations and exposure times and tailoring disinfection strategies to specific HuNoV strains, public health interventions can be enhanced, reducing the global burden of HuNoV-related gastroenteritis. The integration of advanced statistical and machine learning methods in this study sets a precedent for future research, emphasizing the value of computational tools in addressing complex biological questions and improving public health outcomes.

## Data Availability

No datasets were generated or analyzed during the current study.

## References

[CR1] Alvarez, M. E., & O’Brien, R. T. (1982). Effects of chlorine concentration on the structure of poliovirus. *Applied and Environmental Microbiology,**43*(1), 237–239. 10.1128/aem.43.1.237-239.1982 6275791 10.1128/aem.43.1.237-239.1982 PMC241806

[CR2] Bates, D., Mächler, M., Bolker, B., & Walker, S. (2015). Fitting linear mixed-effects models using lme4. *Journal of Statistical Software*. 10.18637/jss.v067.i01

[CR3] Borchardt, M. A., Kieke, B. A., Jr., Spencer, S. K., Lambertini, E., Burch, T. R., & Loge, F. J. (2023). Community intervention trial for estimating risk of acute gastrointestinal illness from groundwater-supplied non-disinfected drinking water. *Journal of Water and Health,**21*(9), 1209–1227. 10.2166/wh.2023.071 37756190 10.2166/wh.2023.071

[CR4] Breiman, L. (2001). Random Forests. *Machine Learning,**45*(1), 5–32. 10.1023/a:1010933404324

[CR5] Buesa, J., & Rodríguez-Díaz, J. (2016). Norovirus infection: Why are the genogroup II genotype 4 strains so persistent in the population? *Future Virology,**11*(11), 711–714. 10.2217/fvl-2016-0101

[CR6] Bull, R. A., Tu, E. T., McIver, C. J., Rawlinson, W. D., & White, P. A. (2006). Emergence of a new norovirus genotype II.4 variant associated with global outbreaks of gastroenteritis. *Journal of Clinical Microbiology,**44*(2), 327–333. 10.1128/JCM.44.2.327-333.2006 16455879 10.1128/JCM.44.2.327-333.2006 PMC1392656

[CR7] Bustin, S. A., Benes, V., Garson, J. A., Hellemans, J., Huggett, J., Kubista, M., Mueller, R., Nolan, T., Pfaffl, M. W., Shipley, G. L., Vandesompele, J., & Wittwer, C. T. (2009). The MIQE guidelines: Minimum information for publication of quantitative real-time PCR experiments. *Clinical Chemistry,**55*(4), 611–622. 10.1373/clinchem.2008.112797 19246619 10.1373/clinchem.2008.112797

[CR8] Canty, A., & Ripley B. D. (2024). boot: Bootstrap Functions. R package version. 1.3–30. https://CRAN.R-project.org/package=boot

[CR9] Chandran, S., & Gibson, K. E. (2024). Improving the detection and understanding of infectious human norovirus in food and water matrices: A review of methods and emerging models. *Viruses,**16*(5), 776. 10.3390/v16050776 38793656 10.3390/v16050776 PMC11125872

[CR10] Chhabra, G., Vashisht, V., & Ranjan, J. (2017). A comparison of multiple imputation methods for data with missing values. *Indian Journal of Science and Technology*. 10.17485/ijst/2017/v10i19/110646

[CR11] Chhabra, P., de Graaf, M., Parra, G. I., Chan, M. C. W., Green, K., Martella, V., et al. (2019). Updated classification of norovirus genogroups and genotypes. *Journal of General Virology,**100*(10), 1393–1406. 10.1099/jgv.0.001318 31483239 10.1099/jgv.0.001318 PMC7011714

[CR71] Choi, J. M., Hutson, A. M., Estes, M. K., & Prasad, B. V. (2008). Atomic resolution structural characterization of recognition of histo-blood group antigens by Norwalk virus. *Proceedings of the National Academy of Sciences*, *105*(27), 9175–9180. 10.1073/pnas.0803275105 10.1073/pnas.0803275105 PMC2453692 18599458

[CR12] Clark, R. M. (1968). A mathematical model of the kinetics of viral devitalization. *Mathematical Biosciences,**2*(3–4), 413–423. 10.1016/0025-5564(68)90026-6

[CR13] Cook, N., Knight, A., & Richards, G. P. (2016). Persistence and elimination of human norovirus in food and on food contact surfaces: A critical review. *Journal of Food Protection,**79*(7), 1273–1294. 10.4315/0362-028x.jfp-15-570 27357051 10.4315/0362-028X.JFP-15-570

[CR14] Costantini, V., Morantz, E. K., Browne, H., Ettayebi, K., Zeng, X. L., Atmar, R. L., Estes, M. K., & Vinjé, J. (2018). Human norovirus replication in human intestinal enteroids as model to evaluate virus inactivation. *Emerging Infectious Diseases,**24*(8), 1453–1464. 10.3201/eid2408.180126 30014841 10.3201/eid2408.180126 PMC6056096

[CR15] Davison, A. C., & Hinkley, D. V. (1997). *Bootstrap Methods and Their Application*. 10.1017/cbo9780511802843

[CR16] Djebbi-Simmons, D., Alhejaili, M., Janes, M., King, J., & Xu, W. (2020). Survival and inactivation of human norovirus GII.4 sydney on commonly touched airplane cabin surfaces. *AIMS Public Health,**7*(3), 574–586. 10.3934/publichealth.2020046 32968679 10.3934/publichealth.2020046 PMC7505796

[CR17] Dunkin, N., Weng, S., Jacangelo, J. G., & Schwab, K. J. (2017). Inactivation of human norovirus genogroups I and II and surrogates by free chlorine in postharvest leafy green wash water. *Applied and Environmental Microbiology*. 10.1128/aem.01457-17 28887415 10.1128/AEM.01457-17 PMC5666131

[CR18] Escudero-Abarca, B. I., Goulter, R. M., Bradshaw, J., Faircloth, J., Leslie, R. A., Manuel, C. S., et al. (2022). Efficacy of an alcohol-based surface disinfectant formulation against human norovirus. *Journal of Applied Microbiology,**132*(5), 3590–3600. 10.1111/jam.15479 35137492 10.1111/jam.15479 PMC9306916

[CR72] Ettayebi, K., Crawford, S. E., Murakami, K., Broughman, J. R., Karandikar, U., Tenge, V. R., Neill, F. H., Blutt, S. E., Zeng, X. L., Qu, L., Kou, B., Opekun, A. R., Burrin, D., Graham, D. Y., Ramani, S., Atmar, R. L., & Estes, M. K. (2016). Replication of human noroviruses in stem cell–derived human enteroids. *Science*, *353*(6306), 1387–1393. 10.1126/science.aaf5211 27562956 10.1126/science.aaf5211 PMC5305121

[CR19] Ettayebi, K., Salmen, W., Imai, K., Hagi, A., Neill, F. H., Atmar, R. L., et al. (2022). Antiviral activity of olanexidine-containing hand rub against human noroviruses. *Mbio*. 10.1128/mbio.02848-21 35297675 10.1128/mbio.02848-21 PMC9040745

[CR20] Farahmand, M., Moghoofei, M., Dorost, A., Shoja, Z., Ghorbani, S., Kiani, S. J., Khales, P., Esteghamati, A., Sayyahfar, S., Jafarzadeh, M., Minaeian, S., Khanaliha, K., Naghdalipour, M., & Tavakoli, A. (2022). Global prevalence and genotype distribution of norovirus infection in children with gastroenteritis: A meta-analysis on 6 years of research from 2015 to 2020. *Reviews in Medical Virology,**32*(1), e2237. 10.1002/rmv.2237 33793023 10.1002/rmv.2237

[CR21] Fávero, L. P., Duarte, A., & Santos, H. P. (2024). A new computational algorithm for assessing overdispersion and zero-inflation in machine learning count models with python. *Computers,**13*(4), 88. 10.3390/computers13040088

[CR22] Gallandat, K., Kolus, R. C., Julian, T. R., & Lantagne, D. S. (2021). A systematic review of chlorine-based surface disinfection efficacy to inform recommendations for low-resource outbreak settings. *American Journal of Infection Control,**49*(1), 90–103. 10.1016/j.ajic.2020.05.014 32442652 10.1016/j.ajic.2020.05.014 PMC7236738

[CR23] Gerba, C. P., & Betancourt, W. Q. (2017). Viral aggregation: Impact on virus behavior in the environment. *Environmental Science & Technology,**51*(13), 7318–7325. 10.1016/10.1021/acs.est.6b05835 28599109 10.1021/acs.est.6b05835

[CR24] Gibson, K. E., Guo, Y., Schissler, J. T., Opryszko, M. C., & Schwab, K. J. (2011). Evaluation of human enteric viruses in surface water and drinking water resources in southern Ghana. *The American Journal of Tropical Medicine and Hygiene,**84*(1), 20–29. 10.4269/ajtmh.2011.10-0389 21212196 10.4269/ajtmh.2011.10-0389 PMC3005515

[CR25] Gibson, K. E., Schwab, K. J., Spencer, S. K., & Borchardt, M. A. (2012). Measuring and mitigating inhibition during quantitative real time PCR analysis of viral nucleic acid extracts from large-volume environmental water samples. *Water Research,**46*(13), 4281–4291. 10.1016/j.watres.2012.04.030 22673345 10.1016/j.watres.2012.04.030

[CR26] Goda, H., Nakayama-Imaohji, H., Yamaoka, H., Tada, A., Nagao, T., Fujisawa, T., et al. (2022). Inactivation of human norovirus by chlorous acid water, a novel chlorine-based disinfectant. *Journal of Infection and Chemotherapy,**28*(1), 67–72. 10.1016/j.jiac.2021.10.001 34635450 10.1016/j.jiac.2021.10.001

[CR27] Graves, S., Piepho, H.P., & Selzer, L. (2019). multcompView: Visualizations of Paired Comparisons. R package version 0.1–8. https://CRAN.R-project.org/package=multcompView

[CR73] Hardy, M. E. (2005). Norovirus protein structure and function. *FEMS Microbiology Letters*, *253*(1), 1–8. 10.1016/j.femsle.2005.08.031 16168575 10.1016/j.femsle.2005.08.031

[CR28] Hayashi, T., Kobayashi, S., Hirano, J., & Murakami, K. (2024). Human norovirus cultivation systems and their use in antiviral research. *Journal of Virology*. 10.1128/jvi.01663-23 38470106 10.1128/jvi.01663-23 PMC11019851

[CR29] Hoff, J. C., & Akin, E. W. (1986). Microbial resistance to disinfectants: Mechanisms and significance. *Environmental Health Perspectives,**69*, 7–13. 10.1289/ehp.86697 3816738 10.1289/ehp.86697 PMC1474323

[CR30] Hothorn, T., Bretz, F., & Westfall, P. (2008). Simultaneous inference in general parametric models. *Biometrical Journal,**50*, 346–363. 10.1002/bimj.200810425 18481363 10.1002/bimj.200810425

[CR31] Kadoya, S., Urayama, S., Nunoura, T., Hirai, M., Takaki, Y., Kitajima, M., et al. (2022). The intrapopulation genetic diversity of RNA virus may influence the sensitivity of chlorine disinfection. *Frontiers in Microbiology*. 10.3389/fmicb.2022.839513 35668760 10.3389/fmicb.2022.839513 PMC9163991

[CR32] Kageyama, T., Kojima, S., Shinohara, M., Uchida, K., Fukushi, S., Hoshino, F. B., et al. (2003). Broadly reactive and highly sensitive assay for Norwalk-like viruses based on real-time quantitative reverse transcription-PCR. *Journal of Clinical Microbiology,**41*(4), 1548–1557. 10.1128/jcm.41.4.1548-1557.2003 12682144 10.1128/JCM.41.4.1548-1557.2003 PMC153860

[CR33] Kassambara A. (2020). ggpubr: ‘ggplot2’ Based Publication Ready Plots. R package version 0.4.0. https://CRAN.R-project.org/package=ggpubr

[CR34] Kassambara A. (2021). rstatix: Pipe-Friendly Framework for Basic Statistical Tests. R package version 0.7.0. https://CRAN.R-project.org/package=rstatix

[CR35] Kelly, D., Allen, D. J., Akello, J. O., Hau, S., Iturriza-Gómara, M., & NoVAS Study Consortium. (2022). A comparison of two methods for detection of norovirus RNA in environmental swab samples. *Applied Microbiology,**2*(3), 460–469. 10.3390/applmicrobiol2030035

[CR36] Keswick, B. H., Satterwhite, T. K., Johnson, P. C., DuPont, H. L., Secor, S. L., Bitsura, J. A., et al. (1985). Inactivation of Norwalk virus in drinking water by chlorine. *Applied and Environmental Microbiology,**50*(2), 261–264. 10.1128/aem.50.2.261-264.1985 2996421 10.1128/aem.50.2.261-264.1985 PMC238613

[CR37] Knight, A., Li, D., Uyttendaele, M., & Jaykus, L.-A. (2012). A critical review of methods for detecting human noroviruses and predicting their infectivity. *Critical Reviews in Microbiology,**39*(3), 295–309. 10.3109/1040841x.2012.709820 22900992 10.3109/1040841X.2012.709820

[CR38] Kuhn, M. (2008). Building predictive models in r using the caret package. *Journal of Statistical Software*. 10.18637/jss.v028.i05

[CR39] Kuznetsova, A., Brockhoff, P. B., & Christensen, R. H. (2017). lmertest package: Tests in linear mixed effects models. *Journal of Statistical Software*. 10.18637/jss.v082.i13

[CR74] Lambertini, E., Spencer, S. K., Bertz, P. D., Loge, F. J., Kieke, B. A., & Borchardt, M. A. (2008). Concentration of enteroviruses, adenoviruses, and noroviruses from drinking water by use of glass wool filters. *Applied and Environmental Microbiology*, *74*(10), 2990–2996. 10.1128/AEM.02246-07 18359827 10.1128/AEM.02246-07 PMC2394941

[CR40] Length, R.V., Buerkner, P., Herve, M., Love, J., Miguez, F., Riebl, H., Singmann, H. (2021). emmeans: Estimated Marginal Means, aka Least-Squares Means. R package version 1.7.5. https://CRAN.R-project.org/package=emmeans

[CR41] Liang, Y., Wang, W. B., Zhang, J., Hou, J. W., Tang, F., Zhang, X. F., et al. (2021). Evolution of the interactions between GII.4 noroviruses and histo-blood group antigens: Insights from experimental and computational studies. *PLOS Pathogens*. 10.1371/journal.ppat.1009745 34252166 10.1371/journal.ppat.1009745 PMC8297928

[CR42] van der Loo, M. (2022). simputation: Simple Imputation. R package version 0.2.8. https://CRAN.R-project.org/package=simputation

[CR43] Lopman, B. A., Steele, D., Kirkwood, C. D., & Parashar, U. D. (2016). The vast and varied global burden of norovirus: Prospects for prevention and control. *PLOS Medicine*. 10.1371/journal.pmed.1001999 27115709 10.1371/journal.pmed.1001999 PMC4846155

[CR44] Manuel, C. S., Moore, M. D., & Jaykus, L. A. (2016). Efficacy of a disinfectant containing silver dihydrogen citrate against GI.6 and GII.4 human norovirus. *Journal of Applied Microbiology,**122*(1), 78–86. 10.1111/jam.13331 27775827 10.1111/jam.13331

[CR45] Mattle, M. J., Crouzy, B., Brennecke, M., Wigginton, K. R., Perona, P., & Kohn, T. (2011). Impact of virus aggregation on inactivation by peracetic acid and implications for other disinfectants. *Environmental Science & Technology,**45*(18), 7710–7717. 21819042 10.1021/es201633s

[CR46] Montazeri, N., Manuel, C., Moorman, E., Khatiwada, J. R., Williams, L. L., & Jaykus, L. A. (2017). Virucidal activity of fogged chlorine dioxide- and hydrogen peroxide-based disinfectants against human norovirus and its surrogate, feline calicivirus, on hard-to-reach surfaces. *Frontiers in Microbiology*. 10.3389/fmicb.2017.01031 28642746 10.3389/fmicb.2017.01031 PMC5462988

[CR47] Monteiro, S., & Santos, R. (2017). Enzymatic and viability RT-qPCR assays for evaluation of enterovirus, hepatitis A virus and norovirus inactivation: Implications for public health risk assessment. *Journal of Applied Microbiology,**124*(4), 965–976. 10.1111/jam.13568 28833965 10.1111/jam.13568

[CR48] Nowak, P., Topping, J. R., Bellamy, K., Fotheringham, V., Gray, J. J., Golding, J. P., et al. (2011). Virolysis of Feline Calicivirus and human GII.4 norovirus following chlorine exposure under standardized light soil disinfection conditions. *Journal of Food Protection,**74*(12), 2113–2118. 10.4315/0362-028x.jfp-11-087 22186052 10.4315/0362-028X.JFP-11-087

[CR49] Nuanualsuwan, S., & Cliver, D. O. (2003). Capsid functions of inactivated human picornaviruses and Feline Calicivirus. *Applied and Environmental Microbiology,**69*(1), 350–357. 10.1128/aem.69.1.350-357.2003alv 12514015 10.1128/AEM.69.1.350-357.2003 PMC152381

[CR50] Okada, M., Ogawa, T., Kaiho, I., & Shinozaki, K. (2005). Genetic analysis of noroviruses in Chiba Prefecture, Japan, between 1999 and 2004. *Journal of Clinical Microbiology,**43*(9), 4391–4401. 10.1128/JCM.43.9.4391-4401.2005 16145082 10.1128/JCM.43.9.4391-4401.2005 PMC1234054

[CR51] Park, G. W., Collins, N., Barclay, L., Hu, L., Prasad, B. V., Lopman, B. A., & Vinjé, J. (2016). Strain-specific virolysis patterns of human noroviruses in response to alcohols. *PLoS ONE*. 10.1371/journal.pone.0157787 27337036 10.1371/journal.pone.0157787 PMC4919085

[CR52] R Core Team. (2021). R: A language and environment for statistical computing. R Foundation for Statistical Computing, Vienna, Austria. https://www.R-project.org/.

[CR53] Ridgeway, G. (2019). gbm: Generalized Boosted Regression Models. R package version 2.1.9. https://CRAN.R-project.org/package=gbm

[CR75] Scallan, E., Hoekstra, R. M., Angulo, F. J., Tauxe, R. V., Widdowson, M. A., Roy, S. L., Jones, J. L., & Griffin, P. M. (2011). Foodborne illness acquired in the United States—major pathogens. *Emerging Infectious Diseases*, *17*(1), 7–15. 10.3201/eid1701.p11101 21192848 10.3201/eid1701.P11101 PMC3375761

[CR54] Shin, G.-A., & Sobsey, M. D. (2008). Inactivation of norovirus by chlorine disinfection of water. *Water Research,**42*(17), 4562–4568. 10.1016/j.watres.2008.08.001 18760818 10.1016/j.watres.2008.08.001

[CR55] Siebenga, J. J., Vennema, H., Zheng, D. P., Vinjé, J., Lee, B. E., Pang, X. L., Ho, E. C., Lim, W., Choudekar, A., Broor, S., Halperin, T., Rasool, N. B., Hewitt, J., Greening, G. E., Jin, M., Duan, Z. J., Lucero, Y., O’Ryan, M., Hoehne, M., … Koopmans, M. (2009). Norovirus illness is a global problem: emergence and spread of norovirus GII.4 variants, 2001–2007. *The Journal of Infectious Diseases,**200*(5), 802–812. 10.1086/605127 19627248 10.1086/605127

[CR56] Svec, D., Tichopad, A., Novosadova, V., Pfaffl, M. W., & Kubista, M. (2015). How good is a PCR efficiency estimate: Recommendations for precise and robust qPCR efficiency assessments. *Biomolecular Detection and Quantification,**3*, 9–16. 10.1016/j.bdq.2015.01.005 27077029 10.1016/j.bdq.2015.01.005 PMC4822216

[CR57] Tierney, N., & Cook, D. (2023). Expanding Tidy data principles to facilitate missing data exploration, visualization and assessment of imputations. *Journal of Statistical Software*. 10.18637/jss.v105.i07

[CR58] Torii, S., Corre, M.-H., Miura, F., Itamochi, M., Haga, K., Katayama, K., et al. (2022). Genotype-dependent kinetics of enterovirus inactivation by free chlorine and ultraviolet (UV) irradiation. *Water Research,**220*, 118712. 10.1016/j.watres.2022.118712 35691190 10.1016/j.watres.2022.118712

[CR59] Torrey, J., von Gunten, U., & Kohn, T. (2019). Differences in viral disinfection mechanisms as revealed by quantitative transfection of echovirus 11 genomes. *Applied and Environmental Microbiology*. 10.1128/aem.00961-19 31076437 10.1128/AEM.00961-19 PMC6606871

[CR60] Trujillo, A. A., McCaustland, K. A., Zheng, D.-P., Hadley, L. A., Vaughn, G., Adams, S. M., et al. (2006). Use of taqman real-time reverse transcription-PCR for rapid detection, quantification, and typing of norovirus. *Journal of Clinical Microbiology,**44*(4), 1405–1412. 10.1128/jcm.44.4.1405-1412.2006 16597869 10.1128/JCM.44.4.1405-1412.2006 PMC1448641

[CR61] Tung, G., Macinga, D., Arbogast, J., & Jaykus, L.-A. (2013). Efficacy of commonly used disinfectants for inactivation of human noroviruses and their surrogates. *Journal of Food Protection,**76*(7), 1210–1217. 10.4315/0362-028x.jfp-12-532 23834796 10.4315/0362-028X.JFP-12-532

[CR62] Tung-Thompson, G., Gentry-Shields, J., Fraser, A., & Jaykus, L. A. (2014). Persistence of human norovirus RT-qPCR signals in simulated gastric fluid. *Food and Environmental Virology,**7*(1), 32–40. 10.1007/s12560-014-9170-4 25344785 10.1007/s12560-014-9170-4

[CR63] United States Centers for Disease Control Prevention (CDC). (2011). Updated norovirus outbreak management and disease prevention guidelines. *MMWR—Recommendations and Reports,**60*, 1–18. 21368741

[CR64] van Buuren, S., & Groothuis-Oudshoorn, K. (2011). mice: Multivariate imputation by chained equations in *R*. *Journal of Statistical Software*. 10.18637/jss.v045.i03

[CR65] Warnes, G.R., Bolker, B., Gorjanc, G., Grothendieck, G., Korosec, A., Lumley, T., MacQueen, D., Magnusson, A., Rogers, J. (2022). gdata: Various R Programming Tools for Data Manipulation. R package version 2.18.0.1. https://CRAN.R-project.org/package=gdata

[CR66] Wickham, H. (2016). *ggplot2: Elegant graphics for data analysis*. New York: Springer-Verlag.

[CR67] Wickham, H., Averick, M., Bryan, J., Chang, W., McGowan, L., François, R., Grolemund, G., Hayes, A., Henry, L., Hester, J., Kuhn, M., Pedersen, T., Miller, E., Bache, S., Müller, K., Ooms, J., Robinson, D., Seidel, D., Spinu, V., & Yutani, H. (2019). Welcome to the Tidyverse. *Journal of Open Source Software*. 10.21105/joss.01686

[CR68] Wigginton, K. R., Pecson, B. M., Sigstam, T., Bosshard, F., & Kohn, T. (2012). Virus inactivation mechanisms: Impact of disinfectants on virus function and structural integrity. *Environmental Science & Technology,**46*(21), 12069–12078. 10.1021/es3029473 23098102 10.1021/es3029473

[CR69] Zhang, X., Chen, C., Du, Y., Yan, D., Jiang, D., Liu, X., Yang, M., Ding, C., Lan, L., Hecht, R., & Yang, S. (2022). Global burden and trends of norovirus-associated diseases from 1990 to 2019: An observational trend study. *Frontiers in Public Health*. 10.3389/fpubh.2022.905172 35784210 10.3389/fpubh.2022.905172 PMC9247406

[CR70] Zhang, P., Hao, C., Di, X., Chuizhao, X., Jinsong, L., Guisen, Z., Hui, L., & Zhaojun, D. (2024). Global prevalence of norovirus gastroenteritis after emergence of the GII.4 Sydney 2012 variant: a systematic review and meta-analysis. *Frontiers in Public Health,**12*, 1373322. 38993708 10.3389/fpubh.2024.1373322 PMC11236571

